# Enhancement in heat transfer due to hybrid nanoparticles in MHD flow of Brinkman-type fluids using Caputo fractional derivatives

**DOI:** 10.1038/s41598-022-18110-1

**Published:** 2022-08-18

**Authors:** Nadeem Ahmad Sheikh, Dennis Ling Chuan Ching, Ilyas Khan, Hamzah bin Sakidin

**Affiliations:** 1 Fundamental and Applied Sciences Department, Universiti Teknologi PETRONAS, 32610 Seri Iskandar, Perak Malaysia; 2 Department of Mathematics, College of Science Al-Zulfi, Majmaah University, Al-Majmaah, 11952 Saudi Arabia

**Keywords:** Applied mathematics, Fluid dynamics

## Abstract

The flow of fluid through porous media is of great importance in industry and other physical situations, Darcy’s law is one of the most useful laws to describe such situation, however, the flows through a dense swarm of particles or through a very high porous media cannot be elaborated by this law. To overcome this difficulty, Brinkman proposed a new idea of Brinkman-type fluid in highly porous media. In this study, the Brinkman-type fluid flow is analyzed with hybrid nanoparticles (a hybridized mixture of clay and alumina), suspended in water taken as a base fluid under the effect of an applied magnetic field. The fluid motion is taken inside a vertical channel with heated walls. Free convection is induced due to buoyancy. The momentum and energy equations are written in dimensionless form using the non-dimensional variables. The energy equation is modified to fractional differential equations using the generalized Fourier’s law and the Caputo fractional derivatives. The fractional model is solved using the Laplace and Fourier transformation. Variations in velocity and temperature are shown for various fractional parameter values, as well as charts for the classical model. For the volume fractions of nanoparticles, the temperature distribution increases, with maximum values of hybrid nanoparticles with the highest specified volume fractions. Moreover, due to hybrid nanoparticles, the rate of heat transfer is intensified.

## Introduction

To describe how fluid moves through a porous medium, Darcy's law is one of the physical principles. It was named after Henri Darcy, a French researcher who invented a pipeline system in 1947 to supply water to a French village^1^. In 1949, another scholar added the name Brinkman to Darcy's law as an expansion. They found a new fluid termed Darcy-Brinkman^2^ as a result of this concept, which was developed for transitional flow between boundaries. This model illustrates a fast enough flow through porous medium, with the driving force being kinetic potential, which is connected to fluid velocity, pressure, and gravitational potential. They are a mixture of Darcy's law and the Stokes equations that broaden Darcy's law to account for kinetic energy loss via viscous shear. The Brinkman equation^2,3^ may be used to represent the average fluid flow across an array of sparse, spherical particles. Furthermore, Brinkman's equations explain transitions between two types of flows, fast and slow, where the one happens in channels susceptible to Stokes' equations and the second occurs in a porous medium according to Darcy's law. A molecular dynamics-like simulation approach, called Stokesian dynamics capable of representing the movements and forces of hydrodynamically interacting particles in Stokes flow, is used to discover the basic solution or Green's function for flow of Brinkman-type fluid by Durlofsky and Brady^4^. The asymptotic convergence of the flow of Brinkman type fluid in a static region was analyzed by Manaa et al.^5^. They have considered the three-dimensional Brinkman equation in their analysis. The exact solutions for the MHD flow of Brinkman-type fluid between two boundaries were obtained by Khan et al.^6^. They have generalized the flow model using the Caputo-Fabrizio fractional derivatives operator.

Heat transfer and fluid dynamics analyses rely heavily on the thermal properties of heat transfer fluids^7^. Due to the low thermal conductivity of water, ethylene glycol, and different oils, cooling capabilities using conventional heat transfer fluids have been restricted^8^. About two decades ago, Choi^9^ invented the term "nanofluid" to characterise a problem solver for heat transfer enhancement. Nanofluids are fluids created by scattering solid nanoparticles in a base fluid using the techniques of nanotechnology^10,11^. Adding millimetre or micrometre sized particles to base fluids is one technique to enhance the heat transfer in fluids. When compared to typical heat transfer fluids, many investigations have demonstrated that nanofluid greatly improves heat transfer^12–17^. Hybrid nanofluids, instead, are a more recent type of nanofluid created by dispersing (a) two or more types of nanoparticles in a base fluid, and (b) hybrid (composite) nanoparticles. In one homogenous phase, a hybrid material combines the physical and chemical features of multiple other materials^18^. Nadeem et al.^19^ investigated the hybrid nanofluid make use of the bvp4c technique and the nanoparticles CaO and Al_2_O_3_ in the base fluid. The primary finding of the study was that hybrid nanofluid has a better heat transfer capacity than standard nanofluid. Subhani and Nadeem^20^ investigated the differences between hybrid and basic nanofluids, using the micropolar fluid model to arrive at a numerical solution. They discovered that basic nanofluids transmit heat at a slower rate than hybrid nanofluids. Huminic and Huminic^21^ analysed the entropy generation in the fluid flow with hybrid nanoparticles. According to their research, the impacts of entropy generation on the flow of fluids with hybrid nanoparticles have been discovered to be a significant alternative for basic heat transfer systems. The Caputo-Fabrizio fractional derivatives were utilised by Saqib et al.^22^ to generalize the flow of hybrid nanofluid in a channel. They used the Brinkman-type fluid model in their study and used Laplace transformation techniques to solve the DEs. The flow of a Brinkman-type fluid with hybrid nanoparticles over a vertical plate was explored by Shafie et al.^23^. They generalised the flow model in this work^23^ by applying an unique notion called Atangana-Baleanu fractional derivatives. Ikram et al.^24^ employed the concept of constant proportional Caputo fractional derivatives to generalise MHD flow of Brinkman-type hybrid nanofluid between parallel plates. The model is solved via the Laplace transform approach, using silver and titanium oxide nanoparticles scattered in the base fluid. El-Gazar et al.^25^ used fractional derivatives to describe a solar collector with a hybrid nanofluid and discovered that fractional differential modelling offers more accurate results when compared to experimental data than integer ordered differential systems. Gul et al.^26^ analysed the flow of a hybrid nanofluid by applying the HAM technique to solve differential equations. Adding hybrid nanoparticles to a base fluid, according to the findings, boosts heat transfer rate. The forced convection flow of hybrid nanoparticle was analysed by Benkhedda et al.^27^. They have examined the impacts of the shapes of nanoparticles, the volume fractions of nanoparticles and the Reynold’s number on the heat transfer rate and skin friction. The deposition of thermophoretic particles in a flow of hybrid nanofluid past a revolving tabletop was studied quantitatively by Gowda et al.^28^. For the flow model, the RKF-45 method was utilised to discover solutions. Sathyamurthy et al.^29^ investigated experimentally the flow of hybrid nanofluid in a thermal (PV/T) system. They have concluded that the efficiency of the system is improved by 27.3% using hybrid nanoparticles. Gohar et al.^30^ examined the applications of hybrid nanoparticles in concrete. The MWCNTs and aluminium oxide nanoparticles in the base fluid concrete were used in the investigation, and the phenomena was simulated using fractional derivatives and solved for exact solutions. On the importance and applications of the flow of fluids with hybrid nanoparticles, Eshgarf et al.^31^ have presented a detailed literature survey. The have concluded that ability to transfer heat is way better in hybrid nanofluid compared to conventional fluids.

Differential equations of fractional order are used to describe a broad variety of physical situations. Reimann-Liouville derivatives^32–36^, Caputo derivatives^37,38^, Caputo Fabrizio derivatives^39,40^, and Atangana-Baleanu fractional derivatives^41–43^ are examples of fractional order derivatives. Akgül et al.^44^ used three fractional differential definitions in their study to provide analytical and approximated solutions for financial/economic models centred on market equilibrium and option pricing. For the investigation of microbial survival and population growth modelling, Ozarslan^45^ employed non-integer order derivatives. Gdawiec, et al.^46^ updated Newton's iterative approach by substituting fractional differential operators for derivatives. Arshad et al.^47^ looked at the dynamical model for HIV (CD4 + T), taking fractional derivatives into account. Alshabanat et al.^48^ used fractional derivatives to analyse RC electric circuits and identified numerical solutions. Fractional derivatives have been employed by many researchers in the field of fluid dynamics to model flows. Song et al.^49^ used the fractional derivatives to modify the model for MHD flow of second grade fluid over an infinite plate. The flow is studied under the effects pf porous media and heat transfer. Using two different approaches of fractional derivatives, Borah et al.^50^ generalized the MHD flow of second grade fluid with heat and mass transfer and have obtained numerical solutions. Shahrim et al.^51^ analytically solved the fractional model of Casson fluid, the flow was induced due to the accelerated plate. The time dependant bioconvective flow was analysed by Arafa et al.^52^ using the Atangana-Baleanu fractional derivatives. They have used the numerical scheme to solve the equations. Moosavi et al.^53^ studied the convective flow of fractional Maxwell fluid using a numerical scheme, the flow was considered over a backward-facing step.

Keeping in mind the above literature survey, this study focuses on the flow Brinkman-type fluid model with heat transfer and hybrid nanoparticles. The flow model after non-dimensionalization is generalized using the modified Fourier’s law and the fractional derivatives operator, namely, the Caputo fractional operator. The generalized model is then solved using the integral transformations for exact solutions. The flow profile and the temperature distribution are drawn and shown in tables.

## Mathematical formulation

**Figure 1:**
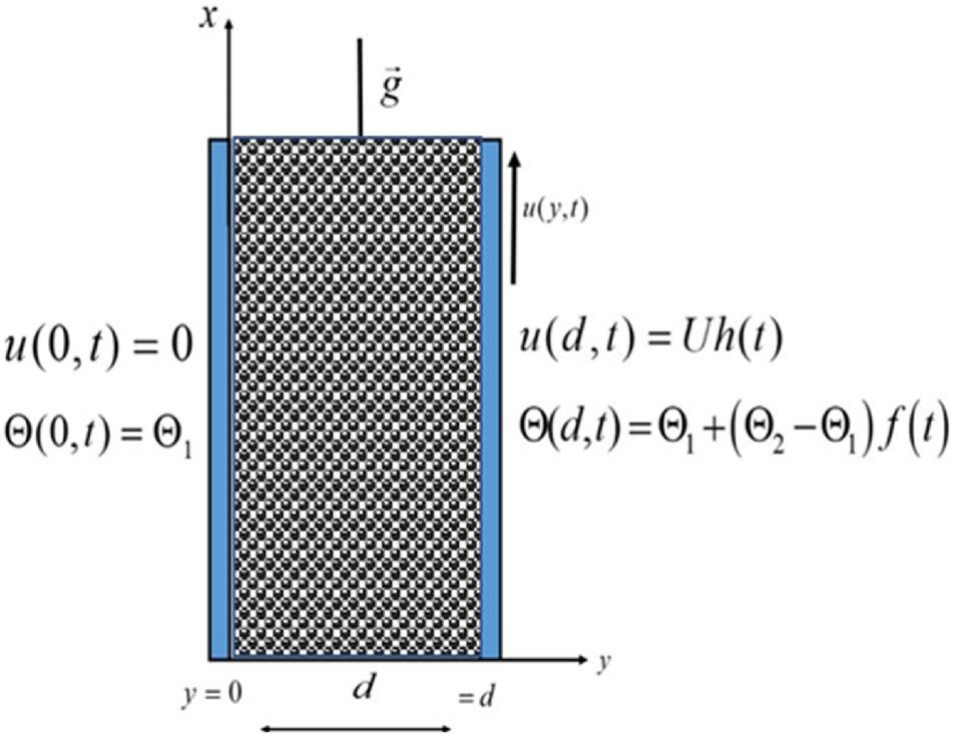
Schematic diagram of the flow.

We have considered the motion of Brinkman-type hybrid nanofluid ia a vertical channel. The flow is assumed to be in the direction of $x$ -axis while the $y$-axis is taken perpendicular to the plates. With ambient temperature $\Theta_{1}$, both the fluid and plates are at rest when $t \le 0$. At $t = 0^{ + }$, the plate at $y = d$ begin to move in its own plane with velocity. At $y = d$, the plate temperature level raised to $\Theta_{1} + \left( {\Theta_{2} - \Theta_{1} } \right)f\left( t \right)$ with time $t.$ The momentum and energy equations for the flow of a Brinkman-type hybrid nanofluid with physical initial and boundary conditions as shown in Fig. 1 are as follows:

1 $$ \rho_{hnf} \frac{\partial u(y,t)}{{\partial t}} + \rho_{hnf} \beta_{r} u(y,t) = \mu_{hnf} \,\frac{{\partial^{2} u(y,t)}}{{\partial y^{2} }} - \sigma_{hnf} B_{0}^{2} u(y,t) + \left( {\rho \beta_{\Theta } } \right)_{hnf} g(\Theta - \Theta_{1} ), $$

2 $$ \left( {\rho C_{p} } \right)_{hnf} \frac{\partial \Theta (y,t)}{{\partial t}} = - \frac{\partial q(y,t)}{{\partial y}}, $$

3 $$ q(y,t) = - k_{hnf} \frac{\partial \Theta (y,t)}{{\partial y}}, $$

with the initial and boundary conditions:

4 $$ \left. {\begin{array}{*{20}l} {u(y,\,0) = 0,} \hfill & {\Theta (y,\,0) = \Theta_{1} ,} \hfill \\ {u(0,\,t) = 0,} \hfill & {\Theta (0,\,t) = \Theta_{1} ,} \hfill \\ {u(d,\,t) = Uh(t),} \hfill & {\Theta (d,\,t) = \Theta_{1} + \left( {\Theta_{2} - \Theta_{1} } \right)f\left( t \right),} \hfill \\ \end{array} } \right\},\quad \quad {\text{for}}\quad f(t) = \Theta_{1} \quad {\text{and}}\quad h(t) = 0\quad {\text{when}}\quad t = 0 $$

where

$$ \rho_{hnf} = \left( {1 - \phi_{hnf} } \right)\rho_{f} + \phi_{s1} \rho_{s1} + \phi_{s2} \rho_{s2} ,\,\,\,\,\,\,\mu_{hnf} = \frac{{\mu_{f} }}{{\left( {1 - \left( {\phi_{s1} + \phi_{s2} } \right)} \right)^{2.5} }},\,\,\,\,\frac{{\sigma_{hnf} }}{{\sigma_{f} }} = 1 + \frac{{3\left( {\frac{{\phi_{s1} \sigma_{s1} + \phi_{s2} \sigma_{s2} }}{{\sigma_{f} }} - \phi_{hnf} } \right)}}{{\left( {\frac{{\phi_{s1} \sigma_{s1} + \phi_{s2} \sigma_{s2} }}{{\phi_{hnf} \sigma_{f} }} + 2} \right) - \left( {\frac{{\phi_{s1} \sigma_{s1} + \phi_{s2} \sigma_{s2} }}{{\sigma_{f} }} - \phi_{hnf} } \right)}}, $$

$$ \left( {\rho \beta_{T} } \right)_{hnf} = \left( {1 - \phi_{hnf} } \right)\left( {\rho \beta_{T} } \right)_{f} + \phi_{s1} \left( {\rho \beta_{T} } \right)_{s1} + \phi_{s2} \left( {\rho \beta_{T} } \right)_{s2} ,\,\,\,\left( {\rho C_{p} } \right)_{hnf} = \left( {1 - \phi_{hnf} } \right)\left( {\rho C_{p} } \right)_{f} + \phi_{s1} \left( {\rho C_{p} } \right)_{s2} + \phi_{s2} \left( {\rho C_{p} } \right)_{s2} $$

where $u$ denotes fluid velocity in the $x$-direction, $\Theta$ represents temperature, $\rho_{hnf}$ represents hybrid nanofluid density, $\mu_{hnf}$ represents dynamic viscosity, $\beta_{r}$ shows Brinkman-type fluid material parameter, $B_{0}$ is a uniform magnetic field^54^, $\beta_{\Theta }$ indicates thermal expansion coefficient^55^, $g$ is acceleration due to gravity, $C_{p}$ denotes fluid specific heat capacity, and $k$ is the thermal conductivity. The thermophysical properties of the nanoparticles and base fluid are taken from^56,57^.

The Buckingham Pi theorem was used to produce non-dimensional variables, which are listed as under.

$$ v = \frac{u}{U}{, }\xi = \frac{y}{d}{, }\tau = \frac{\nu }{{d^{2} }}t{, }\theta = \frac{{\Theta - \Theta_{1} }}{{\Theta_{2} - \Theta_{1} }}{,}\,\delta = \frac{qd}{{k\left( {\Theta_{2} - \Theta_{1} } \right)}}{, }f(\tau ) = f\left( {\frac{{d^{2} }}{\nu }t} \right){,}\,\,\,h(\tau ) = h\left( {\frac{{d^{2} }}{\nu }t} \right){.} $$

Incorporating the above variables into Eqs. (1), (2), (3) and (4) we get:

5 $$ \frac{\partial v(\xi ,\tau )}{{\partial \tau }} = a_{5} \frac{{\partial^{2} v(\xi ,\tau )}}{{\partial \xi^{2} }} - a_{6} v(\xi ,\tau ) + a_{7} \theta (\xi ,\tau ), $$

6 $$ \frac{\partial \theta (\xi ,\tau )}{{\partial \tau }} = - \frac{1}{{a_{8} \Pr }}\frac{\partial \delta (\xi ,\tau )}{{\partial \xi }}, $$

7 $$ \delta (\xi ,\tau ) = - a_{9} \frac{\partial \theta (\xi ,\tau )}{{\partial \xi }} $$

8 $$ \left. {\begin{array}{*{20}l} {v(\xi ,0) = 0,} \hfill & {\theta (\xi ,0) = 0,} \hfill \\ {v(0,\tau ) = 0,} \hfill & {\theta (0,\tau ) = 0,} \hfill \\ {v(1,\tau ) = h(\tau ),} \hfill & {\theta (1,\tau ) = f(\tau ),} \hfill \\ \end{array} } \right\},\quad {\text{for}}\quad f(\tau ) = h(\tau ) = 0\quad {\text{when}}\quad \tau = 0. $$

### Fractional model

To develop a fractional model, the generalized Fourier's law is considered:

9 $$ \delta (\xi ,\tau ) = - {}^{C}\wp_{\tau }^{1 - \alpha } \left( {\frac{\partial \theta (\xi ,\tau )}{{\partial \xi }}} \right);\,\,0 < \alpha \le 1, $$

here $^{C} \wp_{\tau }^{\alpha } \left( . \right)$ is the Caputo derivatives operator, and is defined as:

10 $$ {}^{C}\wp_{t}^{\alpha } Q(y,t) = \frac{1}{{\Gamma \left( {1 - \alpha } \right)}}\int\limits_{0}^{t} {\dot{Q}} (y,s)(t - s)^{ - \alpha } ds = \eta_{\alpha } (t)*\dot{Q}(y,t);\,\,0 < \alpha \le 1, $$

here $\eta_{\alpha } (t) = \frac{{t^{ - \alpha } }}{{\Gamma \left( {1 - \alpha } \right)}}$ is the singular power-law kernel.

Furthermore,

11 $$ L\left\{ {\eta_{\alpha } (t)} \right\} = \frac{1}{{s^{1 - \alpha } }},\,\left\{ {\eta_{1 - \alpha } *\eta_{\alpha } } \right\}\left( t \right) = 1,\,\eta_{0} (t) = L^{ - 1} \left\{ \frac{1}{s} \right\} = 1,\,\eta_{1} (t) = L^{ - 1} \left\{ 1 \right\} = \zeta (t), $$

$L\left\{ . \right\}$ represents the Laplace transform, $\zeta (.)$ is the Dirac's delta function, and $s$ represents the Laplace transform parameter.

Using the properties and Eq. (10), it is simple to demonstrate the following:

12 $$^{C} \wp_{t}^{0} Q(y,t) = Q(y,t) - Q(y,0),\, $$

13 $$^{C} \wp_{t}^{1} Q(y,t) = \frac{\partial Q(y,t)}{{\partial t}} $$

Using Eqs. (6), (7), (9) and (10) we arrived at:

14 $$ \frac{\partial \theta (\xi ,\tau )}{{\partial t}} = \frac{1}{{a_{10} }}{}^{C}\wp_{\tau }^{1 - \alpha } \left( {\frac{{\partial^{2} \theta (\xi ,\tau )}}{{\partial^{2} \xi }}} \right), $$

Call to mind the time fractional integral operator to attain the equivalent form of Eq. (14)

15 $$ \Im_{t}^{\alpha } Q(y,t) = \left( {\eta_{1 - \alpha } *Q} \right)(t) = \frac{1}{\Gamma \left( \alpha \right)}\int\limits_{0}^{t} {Q(y,s)(t - s)^{\alpha - 1} } ds. $$

This describes the inverse operator of $^{C} \wp_{t}^{\alpha } \left( . \right)$. Using the properties from Eq. (11) we have

16 $$ \begin{aligned} \left( {\Im_{t}^{\alpha } \circ {}^{C}\wp_{\tau }^{\alpha } } \right)Q\left( {y,t} \right) & = \Im_{t}^{\alpha } \left( {{}^{C}\wp_{\tau }^{\alpha } Q\left( {y,t} \right)} \right) = \left[ {\eta_{1 - \alpha } *\left( {\eta_{\alpha } *\dot{Q}} \right)} \right]\left( t \right) \\ & = \left[ {\left( {\eta_{1 - \alpha } *\eta_{\alpha } } \right)*\dot{Q}} \right]\left( t \right) = \left[ {1*\dot{Q}} \right](t) = Q(y,t) - Q(y,0), \\ \end{aligned} $$

17 $$ \Rightarrow \left( {\Im_{t}^{\alpha } \circ {}^{C}\wp_{\tau }^{\alpha } } \right)Q\left( {y,t} \right) = Q(y,t)\,\quad if\quad Q\left( {y,0} \right) = 0. $$

Using the property, $\Im_{t}^{1 - \alpha } \dot{Q}\left( {y,t} \right) = \left( {\eta_{\alpha } *\dot{Q}} \right)(t) = {}^{C}\wp_{t}^{\alpha } Q\left( {y,t} \right),$ Eq. (14) can be written as:

18 $$ {}^{C}\wp_{\tau }^{\alpha } \theta (\xi ,\tau ) = \frac{1}{{a_{10} }}\frac{{\partial^{2} \theta (\xi ,\tau )}}{{\partial^{2} \xi }}. $$

## Methodology and solution of the problem

The obtained factional model is solved using the integral transformation techniques, i.e. the Laplace transform and the finite Fourier sine transform^58^.

### Solution of the energy equation

The following mathematical setting is used

19 $$ \chi \left( {\xi ,\tau } \right) = \theta \left( {\xi ,\tau } \right) - \xi f\left( \tau \right), $$

and Eq. (18) takes the form

20 $$ {}^{C}\wp_{\tau }^{\alpha } \chi (\xi ,\tau ) + \xi {}^{C}\wp_{\tau }^{\alpha } f(\tau ) = \frac{1}{{a_{10} }}\frac{{\partial^{2} \chi (\xi ,\tau )}}{{\partial^{2} \xi }}, $$

The initial and boundary conditions are given as under:

21 $$ \chi (\xi ,0) = 0,\,\,\chi (0,\tau ) = 0,\,\,\chi (1,\tau ) = 0. $$

Combinedly use the Laplace and finite Fourier sine transforms yields the following result.

22 $$ \overline{\chi }_{F} (n,s) = s\overline{f}(s)\frac{{\left( { - 1} \right)^{n} }}{n\pi }\frac{{s^{\alpha - 1} }}{{s^{\alpha } + a_{14} }}, $$

By taking the inverse integral transformations of Eq. (22) we get

23 $$ \chi (\xi ,\tau ) = 2\sum\limits_{n = 1}^{\infty } {\frac{{\left( { - 1} \right)^{n} \sin \left( {\xi n\pi } \right)}}{n\pi }} \int\limits_{0}^{\tau } {\dot{f}} \left( {\tau - t} \right)E_{\alpha ,\alpha - 1} \left( { - a_{14} t^{\alpha } } \right)dt, $$

As a result, the solution for energy equation is:

24 $$ \theta \left( {\xi ,\tau } \right) = \chi \left( {\xi ,\tau } \right) + \xi f\left( \tau \right). $$

### Solution of the momentum equation

Apply the Laplace and Fourier transforms to Eq. (5) and using Eq. (8) we have:

25 $$ \begin{aligned} \overline{v}_{F} \left( {n,s} \right) & = \frac{{\left( { - 1} \right)^{n + 1} \overline{h}\left( s \right)}}{n\pi } + \left( {\frac{{a_{12} }}{s} + \frac{{a_{13} }}{{s + a_{11} }}} \right)\frac{{\left( { - 1} \right)^{n} s\overline{h}\left( s \right)}}{n\pi } \\ & \quad + \frac{{a_{7} }}{{s + a_{11} }}\left( {s\overline{f}(s)\frac{{\left( { - 1} \right)^{n} }}{n\pi }\frac{{s^{\alpha - 1} }}{{s^{\alpha } + a_{14} }} + \overline{f}(s)\frac{{\left( { - 1} \right)^{n + 1} }}{n\pi }} \right), \\ \end{aligned} $$

where

$$ \begin{aligned} a_{1} & = \left( {1 - \phi_{hnf} } \right) + \frac{{\phi_{s1} \rho_{s1} + \phi_{s2} \rho_{s2} }}{{\rho_{f} }},\,a_{2} = \frac{1}{{\left( {1 - \left( {\phi_{s1} + \phi_{s2} } \right)} \right)^{2.5} }}, \\ a_{3} & = 1 + \frac{{3\left( {\frac{{\phi_{s1} \sigma_{s1} + \phi_{s2} \sigma_{s2} }}{{\sigma_{f} }} - \phi_{hnf} } \right)}}{{\left( {\frac{{\phi_{s1} \sigma_{s1} + \phi_{s2} \sigma_{s2} }}{{\phi_{hnf} \sigma_{f} }} + 2} \right) - \left( {\frac{{\phi_{s1} \sigma_{s1} + \phi_{s2} \sigma_{s2} }}{{\sigma_{f} }} - \phi_{hnf} } \right)}},\quad a_{4} = \left( {1 - \phi_{hnf} } \right) + \frac{{\phi_{s1} \left( {\rho \beta_{T} } \right)_{s1} + \phi_{s2} \left( {\rho \beta_{T} } \right)_{s2} }}{{\left( {\rho \beta_{T} } \right)_{f} }}, \\ a_{5} & = \frac{{a_{2} }}{{a_{1} }},\quad a_{6} = \frac{{a_{3} M}}{{a_{1} }} - \gamma_{B} ,\quad a_{7} = \frac{{a_{4} Gr}}{{a_{1} }},\quad a_{8} = \left( {1 - \phi_{hnf} } \right) + \frac{{\phi_{s1} \left( {\rho C_{p} } \right)_{s2} + \phi_{s2} \left( {\rho C_{p} } \right)_{s2} }}{{\left( {\rho C_{p} } \right)_{f} }},\, \\ a_{9} & = \frac{{\frac{{\phi_{s1} k_{s1} + \phi_{s2} k_{s2} }}{{\phi_{hnf} }} + 2k_{f} + 2\left( {\phi_{s1} k_{s1} + \phi_{s2} k_{s2} } \right) - 2k_{f} \phi_{hnf} }}{{\frac{{\phi_{s1} k_{s1} + \phi_{s2} k_{s2} }}{{\phi_{hnf} }} + 2k_{f} - \left( {\phi_{s1} k_{s1} + \phi_{s2} k_{s2} } \right) + k_{f} \phi_{hnf} }},\quad a_{10} = \frac{{a_{8} \Pr }}{{a_{9} }},\quad a_{11} = a_{6} + a_{5} \left( {n\pi } \right)^{2} ,\quad a_{12} = \frac{{a_{6} }}{{a_{11} }}\,,\,\, \\ a_{13} & = \frac{{a_{11} - a_{6} }}{{a_{11} }},\quad a_{14} = \frac{{\left( {n\pi } \right)^{2} }}{{a_{10} }}, \\ \end{aligned} $$

where $M = \tfrac{{\sigma_{f} B_{0}^{2} d^{2} }}{{\mu_{f} }}$ is the Hartman number, $\gamma_{B} = \tfrac{{d^{2} \beta_{r} }}{{\nu_{f} }}$ is Brinkman-type fluid parameter,$\;Gr = \tfrac{{gd^{2} \beta_{\Theta } }}{{\nu_{f} U}}\left( {\Theta_{2} - \Theta_{1} } \right)$ is the thermal Grashof number, and $\Pr = \tfrac{{\left( {\rho C_{p} } \right)_{f} \upsilon_{f} }}{{k_{f} }}$ is the Prandtl number.

Taking the inverse Laplace and finite Fourier sine transformations of Eq. (25) we have:

26 $$ \begin{aligned} v\left( {\xi ,\tau } \right) & = h\left( \tau \right)\xi + 2\sum\limits_{n = 1}^{\infty } {\frac{{\left( { - 1} \right)^{n} }}{n\pi }\dot{h}} \left( \tau \right)*\left( {a_{6} H(\tau ) + a_{13} \exp \left( { - a_{11} \tau } \right)} \right)\sin \left( {\xi n\pi } \right) \\ & \quad + 2a_{7} \sum\limits_{n = 1}^{\infty } {\left( {\frac{{\left( { - 1} \right)^{n} }}{n\pi }\exp \left( { - a_{11} \tau } \right)*\left( {\int\limits_{0}^{\tau } {\dot{f}} \left( {\tau - q} \right)E_{\alpha ,\alpha - 1} \left( { - a_{14} q^{\alpha } } \right)dq + f\left( \tau \right)} \right)} \right)} \sin \left( {\xi n\pi } \right) \\ \end{aligned} $$

here the unit step function is presented by $H\left( . \right)$ and the Mittag–Leffler function is symbolized by $E_{a,b} \left( . \right)$^59^.

### Limiting case

For $\phi_{1} = \phi_{2} = \beta_{r} = 0$, the obtained solution is reduced to the solution calculated by Shao et al.^60^ (for $f_{2} (t) = 0$). This shows the validity of the present solutions. For details please see Eq. 52 in^60^.

## Results and discussion

The flow of a hybrid nanofluid including clay and alumina as suspended nanoparticles is examined under the impact of a magnetic field. Model for convective flow of Brinkman-type fluid is generalized using a modified Fourier's law and the Caputo fractional derivatives. The Laplace and Fourier transformation techniques are used to solve the generalized model for exact solutions. For each parameter, closed-form solutions are drawn.

**Figure 2:**
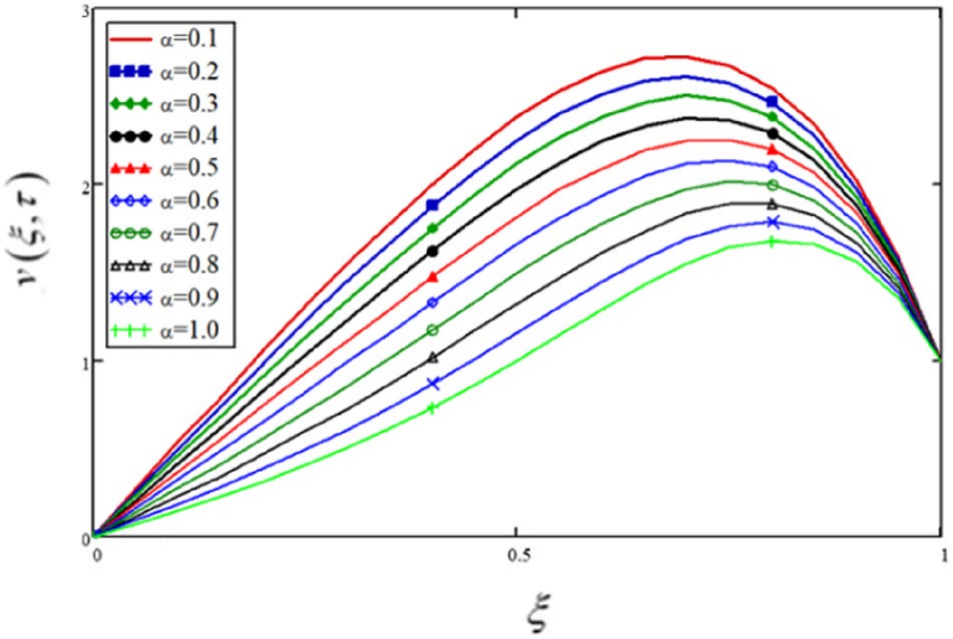
Variations in velocity profile for various values of fractional parameter, the profiles for $\alpha = 0.1,0.2,0.3, \ldots ,1$ ($\alpha = 1$ is the solution for the integer ordered model).

**Figure 3:**
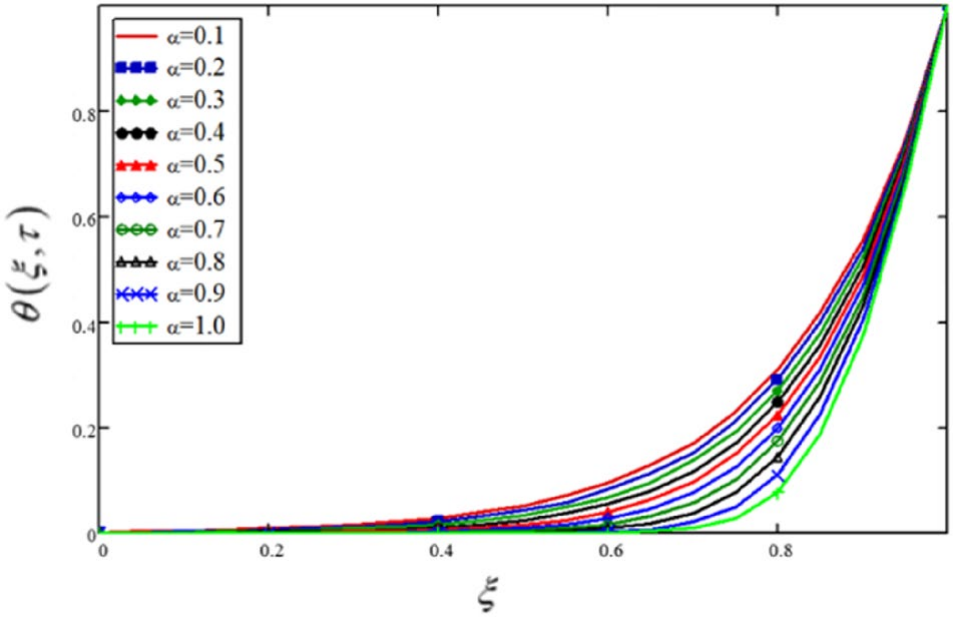
Variations in temperature profile for various values of fractional parameter, the profiles for $\alpha = 0.1,0.2,0.3, \ldots ,1$ ($\alpha = 1$ is the solution for the integer ordered model.

In this study, the fractional parameter is a highly essential and noticeable parameter. As the fractional derivatives are more generalize than the classical derivatives, therefore, fractional derivatives are used to describe the heat transfer. Some other applications are found in fractional-order neurons for parameter estimate, fractional viscoelasticity model, fractional single-phase-lag model of heat conduction, heat convection etc. To show the variation in the flow velocity and the temperature distributions for various values of $\alpha$, Figs. 2 and 3 (also Tables 1, 2) are plotted. The profiles for $\alpha = 0.1,\,0.2,\,0.3,...1$($\alpha = 1$ is the solution for the integer order model) have been drawn, and notable changes have been observed. The physical entities, velocity, and temperature are nonetheless influenced by $\alpha$. This is a fascinating phenomenon that can't be noticed in integer ordered derivatives solutions. Experimenters can utilise these variations for curve fitting and actual results. This behaviour is sometimes referred to as a memory effect in the literature.

**Table 1:** The influence of fractional parameter on the flow velocity.

$\xi$	$v(\xi ,\tau )$ $\alpha = 0.1$	$v(\xi ,\tau )$ $\alpha = 0.2$	$v(\xi ,\tau )$ $\alpha = 0.3$	$v(\xi ,\tau )$ $\alpha = 0.4$	$v(\xi ,\tau )$ $\alpha = 0.5$	$v(\xi ,\tau )$ $\alpha = 0.6$	$v(\xi ,\tau )$ $\alpha = 0.7$	$v(\xi ,\tau )$ $\alpha = 0.8$	$v(\xi ,\tau )$ $\alpha = 0.9$	$v(\xi ,\tau )$ $\alpha = 1.0$
0	0	0	0	0	0	0	0	0	0	0
0.05	0.19	0.181	0.171	0.16	0.149	0.136	0.122	0.108	0.095	0.083
0.1	0.378	0.36	0.341	0.32	0.297	0.272	0.245	0.217	0.19	0.166
0.15	0.563	0.537	0.509	0.478	0.444	0.407	0.367	0.326	0.286	0.251
0.2	0.745	0.71	0.673	0.633	0.59	0.541	0.49	0.436	0.384	0.337
0.25	0.92	0.878	0.833	0.785	0.732	0.674	0.611	0.546	0.483	0.425
0.3	1.088	1.039	0.988	0.933	0.872	0.805	0.733	0.657	0.583	0.516
0.35	1.246	1.192	1.135	1.074	1.006	0.932	0.852	0.768	0.686	0.609
0.4	1.392	1.334	1.273	1.207	1.135	1.055	0.969	0.879	0.789	0.704
0.45	1.525	1.464	1.4	1.331	1.255	1.172	1.082	0.987	0.892	0.802
0.5	1.641	1.579	1.513	1.442	1.365	1.28	1.188	1.092	0.994	0.9
0.55	1.738	1.675	1.609	1.539	1.462	1.378	1.286	1.19	1.093	0.997
0.6	1.811	1.75	1.686	1.617	1.543	1.461	1.373	1.279	1.184	1.09
0.65	1.857	1.799	1.738	1.673	1.603	1.526	1.443	1.355	1.265	1.175
0.7	1.871	1.818	1.762	1.703	1.638	1.568	1.492	1.413	1.331	1.248
0.75	1.85	1.803	1.753	1.701	1.644	1.582	1.516	1.446	1.375	1.302
0.8	1.788	1.748	1.707	1.662	1.615	1.564	1.508	1.451	1.391	1.331
0.85	1.681	1.65	1.617	1.583	1.546	1.506	1.464	1.419	1.373	1.327
0.9	1.524	1.502	1.48	1.457	1.431	1.404	1.375	1.345	1.314	1.282
0.95	1.306	1.295	1.284	1.272	1.259	1.245	1.23	1.215	1.199	1.183
1	1	1	1	1	1	1	1	1	1	1

**Table 2:** The effects of different values of fractional parameters on the temperature distribution.

$\xi$	$\theta (\xi ,\tau )$ $\alpha = 0.1$	$\theta (\xi ,\tau )$ $\alpha = 0.2$	$\theta (\xi ,\tau )$ $\alpha = 0.3$	$\theta (\xi ,\tau )$ $\alpha = 0.4$	$\theta (\xi ,\tau )$ $\alpha = 0.5$	$\theta (\xi ,\tau )$ $\alpha = 0.6$	$\theta (\xi ,\tau )$ $\alpha = 0.7$	$\theta (\xi ,\tau )$ $\alpha = 0.8$	$\theta (\xi ,\tau )$ $\alpha = 0.9$	$\theta (\xi ,\tau )$ $\alpha = 1.0$
0	0	0	0	0	0	0	0	0	0	0
0.05	1.609 × 10^–3^	1.093 × 10^–3^	6.419 × 10^–4^	3.035 × 10^–3^	1.032 × 10^–4^	2.077 × 10^–5^	1.738 × 10^–6^	2.986 × 10^–8^	− 1.791 × 10^–9^	− 3.949 × 10^–9^
0.1	3.363 × 10^–3^	2.306 × 10^–3^	1.374 × 10^–3^	6.667 × 10^–4^	2.372 × 10^–4^	5.193 × 10^–5^	5.129 × 10^–6^	1.256 × 10^–7^	− 3.427 × 10^–9^	− 7.948 × 10^–9^
0.15	5.419 × 10^–3^	3.769 × 10^–3^	2.3 × 10^–3^	1.16 × 10^–3^	4.408 × 10^–4^	1.084 × 10^–4^	1.318 × 10^–5^	4.759 × 10^–7^	− 3.76 × 10^–9^	− 1.205 × 10^–8^
0.2	7.961 × 10^–3^	5.642 × 10^–3^	3.547 × 10^–4^	1.876 × 10^–3^	7.707 × 10^–4^	2.155 × 10^–4^	3.256 × 10^–5^	1.709 × 10^–6^	5.045 × 10^–9^	− 1.63 × 10^–8^
0.25	0.011	8.123 × 10^–3^	5.282 × 10^–3^	2.946 × 10^–3^	1.315 × 10^–3^	4.192 × 10^–4^	7.82 × 10^–4^	5.839 × 10^–6^	7.063 × 10^–8^	− 2.075 × 10^–8^
0.3	0.015	0.011	7.737 × 10^–3^	4.559 × 10^–3^	2.212 × 10^–3^	8.015 × 10^–4^	1.828 × 10^–4^	1.899 × 10^–5^	4.555 × 10^–7^	− 2.493 × 10^–8^
0.35	0.021	0.016	0.011	6.994 × 10^–3^	3.681 × 10^–3^	1.508 × 10^–3^	4.157 × 10^–4^	5.882 × 10^–5^	2.467 × 10^–6^	− 2.141 × 10^–8^
0.4	0.029	0.022	0.016	0.011	6.067 × 10^–3^	2.791 × 10^–3^	9.191 × 10^–4^	1.732 × 10^–4^	1.198 × 10^–5^	7.903 × 10^–8^
0.45	0.039	0.031	0.023	0.016	9.901 × 10^–3^	5.081 × 10^–3^	1.975 × 10^–3^	4.849 × 10^–4^	5.272 × 10^–5^	1.138 × 10^–6^
0.5	0.052	0.043	0.033	0.024	0.016	9.091 × 10^–3^	4.119 × 10^–3^	1.289 × 10^–3^	2.108 × 10^–4^	9.955 × 10^–6^
0.55	0.07	0.059	0.048	0.036	0.026	0.016	8.335 × 10^–3^	3.248 × 10^–3^	7.655 × 10^–4^	7.021 × 10^–5^
0.6	0.095	0.082	0.068	0.054	0.04	0.028	0.016	7.755 × 10^–3^	2.522 × 10^–3^	4.098 × 10^–4^
0.65	0.127	0.112	0.096	0.08	0.063	0.047	0.031	0.018	7.537 × 10^–3^	1.988 × 10^–3^
0.7	0.171	0.154	0.136	0.117	0.098	0.077	0.057	0.037	0.02	8.043 × 10^–3^
0.75	0.23	0.212	0.192	0.171	0.149	0.126	0.101	0.075	0.05	0.027
0.8	0.309	0.29	0.27	0.248	0.225	0.2	0.173	0.143	0.111	0.077
0.85	0.415	0.396	0.377	0.356	0.334	0.31	0.285	0.256	0.223	0.185
0.9	0.556	0.541	0.525	0.508	0.49	0.471	0.451	0.43	0.405	0.377
0.95	0.746	0.737	0.727	0.718	0.709	0.699	0.687	0.676	0.667	0.659
1	1	1	1	1	1	1	1	1	1	1

**Figure 4:**
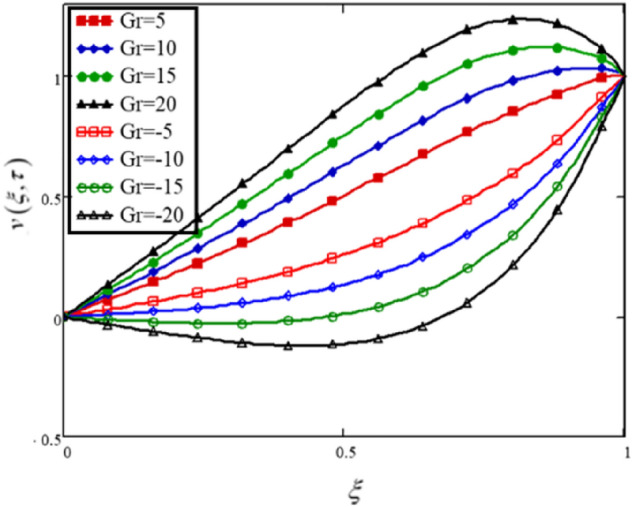
The effect of the Grashof number on the velocity profile of a hybrid nanofluid of the Brinkman type.

In this analysis, the buoyancy component is included in the momentum equation, which after nondimensionalization provides a nondimensional parameter, the Grashof number ($Gr$). Physically, $Gr$ is the ratio of buoyancy forces to viscous forces; thus, increasing $Gr$ values increase buoyancy forces while weakening viscous forces, resulting in increased fluid velocity. Figure 4 and Table 3 show the differences in nanofluid velocity profiles for different values of $Gr$. The heating and cooling of the boundary are represented by the positive and negative values of $Gr$, respectively.

**Table 3:** The effects of Grashof number on the velocity profile of the flow.

$\xi$	$v(\xi ,\tau )$ $Gr = 5$	$v(\xi ,\tau )$ $Gr = 10$	$v(\xi ,\tau )$ $Gr = 15$	$v(\xi ,\tau )$ $Gr = 20$	$v(\xi ,\tau )$ $Gr = - 5$	$v(\xi ,\tau )$ $Gr = - 10$	$v(\xi ,\tau )$ $Gr = - 15$	$v(\xi ,\tau )$ $Gr = - 20$
0	0	0	0	0	0	0	0	0
0.05	0.045	0.058	0.071	0.084	0.019	6.362 × 10^–3^	− 6.584 × 10^–3^	− 0.02
0.1	0.091	0.117	0.143	0.169	0.039	0.013	− 0.013	− 0.039
0.15	0.137	0.176	0.215	0.254	0.059	0.021	− 0.018	− 0.057
0.2	0.185	0.237	0.289	0.34	0.081	0.029	− 0.023	− 0.074
0.25	0.234	0.299	0.363	0.428	0.104	0.04	− 0.025	− 0.09
0.3	0.285	0.362	0.44	0.517	0.13	0.052	− 0.025	− 0.103
0.35	0.337	0.427	0.518	0.608	0.157	0.067	− 0.023	− 0.113
0.4	0.392	0.494	0.596	0.698	0.188	0.086	− 0.016	− 0.119
0.45	0.448	0.562	0.675	0.789	0.221	0.108	− 5.352 × 10^–3^	− 0.119
0.5	0.506	0.63	0.754	0.877	0.259	0.135	0.012	− 0.112
0.55	0.566	0.698	0.83	0.963	0.301	0.169	0.036	− 0.096
0.6	0.626	0.765	0.904	1.043	0.348	0.209	0.07	− 0.069
0.65	0.686	0.829	0.972	1.114	0.401	0.258	0.115	− 0.027
0.7	0.745	0.888	1.031	1.174	0.459	0.317	0.174	0.031
0.75	0.802	0.94	1.078	1.217	0.525	0.386	0.248	0.109
0.8	0.854	0.982	1.11	1.238	0.597	0.469	0.341	0.213
0.85	0.901	1.012	1.123	1.234	0.679	0.568	0.457	0.346
0.9	0.945	1.03	1.115	1.2	0.775	0.69	0.605	0.52
0.95	0.987	1.036	1.084	1.133	0.889	0.841	0.792	0.743
1	1	1	1	1	1	1	1	1

**Figure 5:**
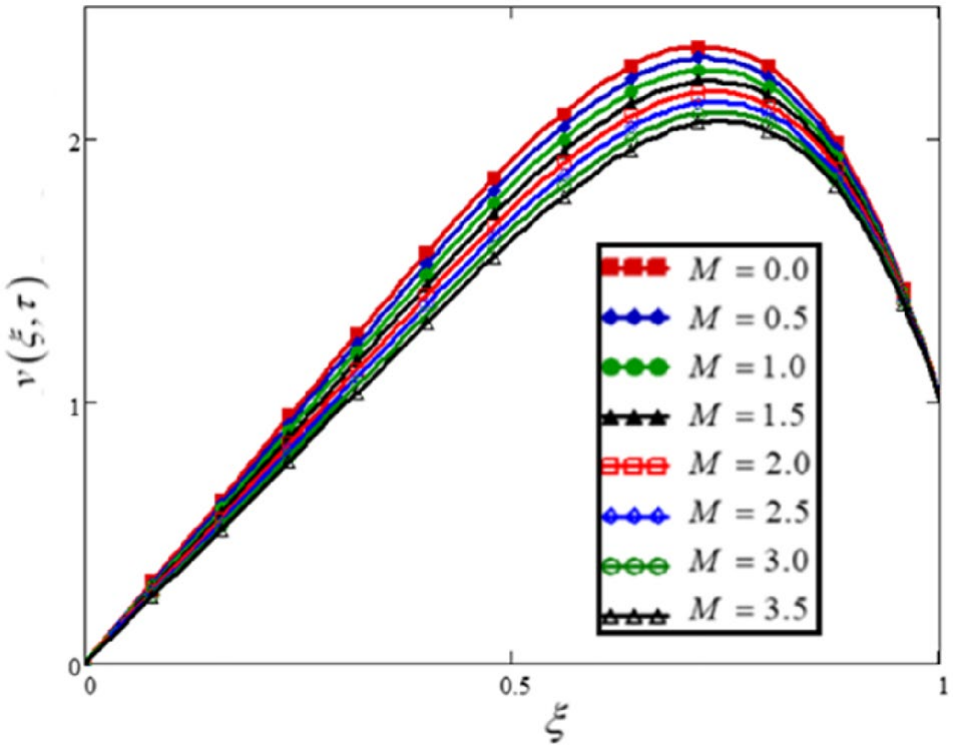
The flow velocity of a Brinkman-type hybrid nanofluid is affected by the Hartman number.

The Lorentz forces, which are flow opposite forces that control the velocity of electrically conducting fluids, are represented by Hartman's number $M$. Figure 5 and Table 4 depict the effect of Hartman's number on fluid velocity. The results show that when the value of $M$ increases, the flow of the hybrid nanofluid decreases.

**Table 4:** For different values of Hartman’s number, the variations in velocity of hybrid nanofluids.

$\xi$	$v(\xi ,\tau )$ $M = 0.0$	$v(\xi ,\tau )$ $M = 0.5$	$v(\xi ,\tau )$ $M = 1.0$	$v(\xi ,\tau )$ $M = 1.5$	$v(\xi ,\tau )$ $M = 2.0$	$v(\xi ,\tau )$ $M = 2.5$		$v(\xi ,\tau )$ $M = 3.0$	$v(\xi ,\tau )$ $M = 3.5M = 3.5$
0	0	0	0	0	0		0	0	0
0.05	0.195	0.189	0.184	0.178	0.173		0.168	0.163	0.158
0.1	0.391	0.379	0.368	0.357	0.347		0.337	0.327	0.318
0.15	0.588	0.57	0.553	0.537	0.521		0.506	0.492	0.478
0.2	0.785	0.761	0.739	0.718	0.697		0.677	0.658	0.64
0.25	0.982	0.953	0.926	0.9	0.874		0.85	0.826	0.804
0.3	1.179	1.145	1.113	1.082	1.052		1.023	0.995	0.969
0.35	1.373	1.335	1.298	1.263	1.228		1.196	1.164	1.134
0.4	1.563	1.52	1.48	1.44	1.403		1.366	1.331	1.298
0.45	1.745	1.699	1.655	1.613	1.572		1.532	1.494	1.458
0.5	1.915	1.866	1.82	1.775	1.731		1.69	1.649	1.611
0.55	2.067	2.017	1.969	1.922	1.877		1.834	1.792	1.752
0.6	2.195	2.145	2.096	2.049	2.003		1.96	1.917	1.876
0.65	2.291	2.241	2.193	2.147	2.102		2.058	2.016	1.976
0.7	2.344	2.297	2.251	2.206	2.163		2.121	2.081	2.042
0.75	2.343	2.299	2.256	2.215	2.176		2.137	2.1	2.064
0.8	2.273	2.235	2.198	2.162	2.127		2.093	2.06	2.029
0.85	2.122	2.091	2.06	2.031	2.003		1.975	1.948	1.922
0.9	1.875	1.853	1.831	1.81	1.79		1.77	1.75	1.732
0.95	1.516	1.504	1.493	1.482	1.471		1.46	1.45	1.44
1	1	1	1	1	1		1	1	1

**Figure 6:**
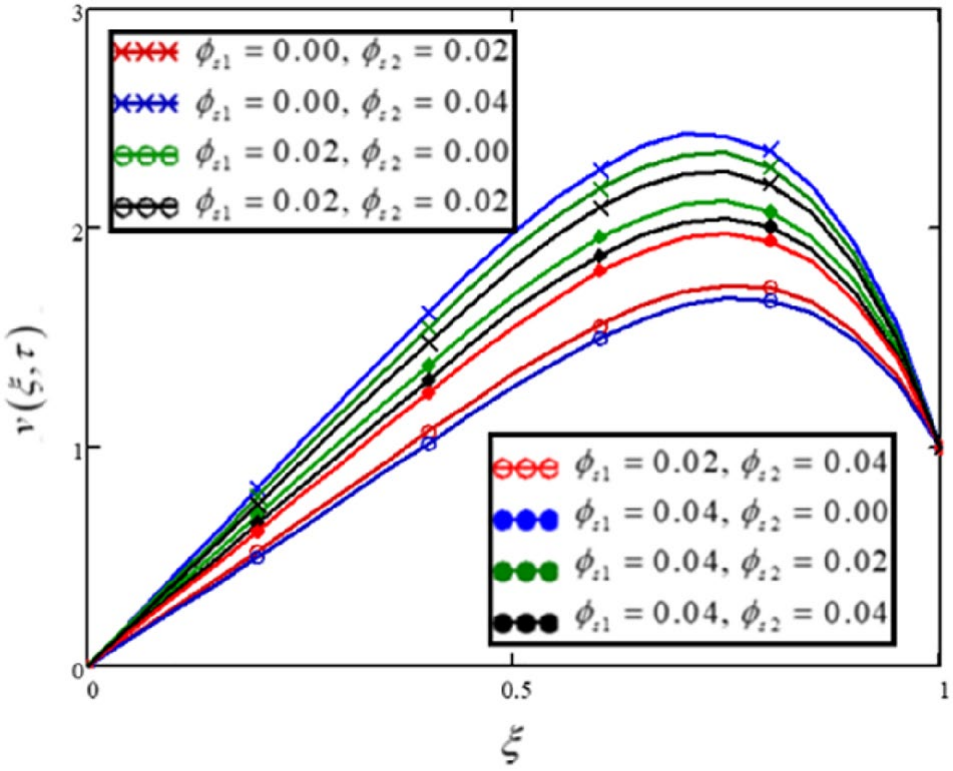
The effect of volume fractions of clay nanoparticles and alumina Al_2_O_3_on the flow profile.

Volume fraction of the solid nanoparticles displays the proportion of the particles in the base fluid. In this analysis, $\phi_{s1}$ and $\phi_{s2}$ are taken as the volume fractions of clay nanoparticles and alumina $Al_{2} O_{3}$ correspondingly. The findings in Fig. 6 and Table 5 depict that the velocity is increasing with the rising values of $\phi_{s1}$ and reducing with the rising values of $\phi_{s2}$.

**Table 5:** The influence of various values of volume fractions of nanoparticles on the velocity profile.

$\xi$	$v(\xi ,\tau )$ $$\begin{gathered} \phi_{s1} = 0.00 \hfill \\ \phi_{s2} = 0.02 \hfill \\ \end{gathered}$$	$v(\xi ,\tau )$ $$\begin{gathered} \phi_{s1} = 0.00 \hfill \\ \phi_{s2} = 0.04 \hfill \\ \end{gathered}$$	$v(\xi ,\tau )$ $$\begin{gathered} \phi_{s1} = 0.02 \hfill \\ \phi_{s2} = 0.00 \hfill \\ \end{gathered}$$	$v(\xi ,\tau )$ $$\begin{gathered} \phi_{s1} = 0.02 \hfill \\ \phi_{s2} = 0.02 \hfill \\ \end{gathered}$$	$v(\xi ,\tau )$ $$\begin{gathered} \phi_{s1} = 0.02 \hfill \\ \phi_{s2} = 0.04 \hfill \\ \end{gathered}$$	$v(\xi ,\tau )$ $$\begin{gathered} \phi_{s1} = 0.04 \hfill \\ \phi_{s2} = 0.00 \hfill \\ \end{gathered}$$	$v(\xi ,\tau )$ $$\begin{gathered} \phi_{s1} = 0.04 \hfill \\ \phi_{s2} = 0.02 \hfill \\ \end{gathered}$$	$v(\xi ,\tau )$ $$\begin{gathered} \phi_{s1} = 0.04 \hfill \\ \phi_{s2} = 0.04 \hfill \\ \end{gathered}$$
0	0	0	0	0	0	0	0	0
0.05	0.112	0.109	0.135	0.132	0.129	0.156	0.153	0.149
0.1	0.223	0.218	0.27	0.264	0.258	0.312	0.305	0.297
0.15	0.334	0.326	0.404	0.395	0.386	0.466	0.456	0.445
0.2	0.444	0.433	0.537	0.525	0.513	0.619	0.605	0.59
0.25	0.553	0.54	0.668	0.653	0.638	0.768	0.751	0.733
0.3	0.66	0.645	0.796	0.778	0.76	0.915	0.894	0.873
0.35	0.764	0.747	0.921	0.9	0.879	1.056	1.033	1.008
0.4	0.866	0.846	1.04	1.017	0.993	1.191	1.164	1.136
0.45	0.963	0.941	1.153	1.128	1.102	1.317	1.288	1.257
0.5	1.054	1.031	1.258	1.23	1.202	1.432	1.401	1.367
0.55	1.137	1.113	1.351	1.322	1.292	1.534	1.5	1.464
0.6	1.21	1.185	1.43	1.4	1.369	1.618	1.582	1.545
0.65	1.27	1.244	1.492	1.461	1.429	1.68	1.644	1.605
0.7	1.314	1.288	1.532	1.501	1.469	1.716	1.679	1.641
0.75	1.339	1.314	1.546	1.516	1.485	1.72	1.684	1.646
0.8	1.34	1.317	1.53	1.501	1.471	1.687	1.652	1.617
0.85	1.315	1.294	1.477	1.451	1.425	1.61	1.579	1.548
0.9	1.26	1.243	1.382	1.362	1.341	1.481	1.457	1.433
0.95	1.163	1.154	1.232	1.22	1.209	1.287	1.274	1.26
1	1	1	1	1	1	1	1	1

**Figure 7:**
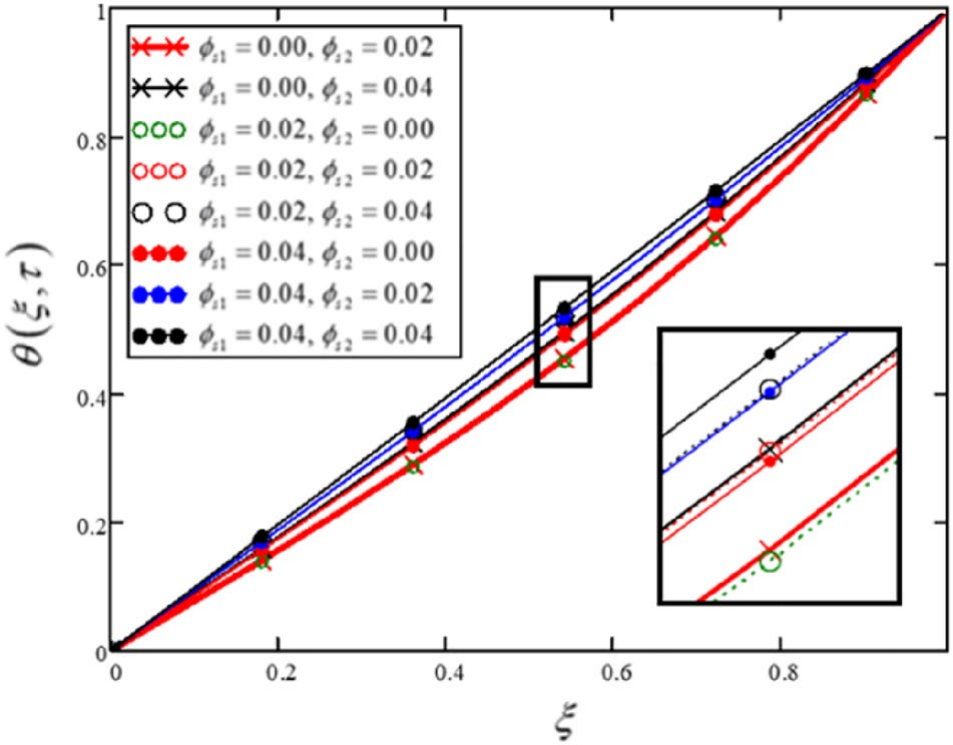
For varied values of volume fractions of hybrid nanoparticles (Clay nanoparticles and Alumina), the temperature profile changes.

The impact of $\phi_{s1}$ and $\phi_{s2}$ on the temperature profile is displayed in Fig. 7 and Table 6. The outcomes shows that for in the non-existence of clay nanoparticles $\left( {\phi_{s1} = 0.0,\,\phi_{s2} = 0.02} \right)$ or Al_2_O_3_ $\left( {\phi_{s1} = 0.02,\,\phi_{s2} = 0.0} \right)$ the heat transfer is lowest and for the greater values of the volume fractions of the nanoparticles $\left( {\phi_{s1} = 0.04,\,\phi_{s2} = 0.04} \right)$ the heat transfer is highest. In common, the results portray that temperature is increasing with the growing values of both $\phi_{s1}$ and $\phi_{s2}$.

**Table 6:** Variations in temperature profile for various values of volume fraction of hybrid nanoparticles (Clay nanoparticles and Alumina).

$\xi$	$\theta (\xi ,\tau )$ $$\begin{gathered} \phi_{s1} = 0.00 \hfill \\ \phi_{s2} = 0.02 \hfill \\ \end{gathered}$$	$\theta (\xi ,\tau )$ $$\begin{gathered} \phi_{s1} = 0.00 \hfill \\ \phi_{s2} = 0.04 \hfill \\ \end{gathered}$$	$\theta (\xi ,\tau )$ $$\begin{gathered} \phi_{s1} = 0.02 \hfill \\ \phi_{s2} = 0.00 \hfill \\ \end{gathered}$$	$\theta (\xi ,\tau )$ $$\begin{gathered} \phi_{s1} = 0.02 \hfill \\ \phi_{s2} = 0.02 \hfill \\ \end{gathered}$$	$\theta (\xi ,\tau )$ $$\begin{gathered} \phi_{s1} = 0.02 \hfill \\ \phi_{s2} = 0.04 \hfill \\ \end{gathered}$$	$\theta (\xi ,\tau )$ $$\begin{gathered} \phi_{s1} = 0.04 \hfill \\ \phi_{s2} = 0.00 \hfill \\ \end{gathered}$$	$\theta (\xi ,\tau )$ $$\begin{gathered} \phi_{s1} = 0.04 \hfill \\ \phi_{s2} = 0.02 \hfill \\ \end{gathered}$$	$\theta (\xi ,\tau )$ $$\begin{gathered} \phi_{s1} = 0.04 \hfill \\ \phi_{s2} = 0.04 \hfill \\ \end{gathered}$$
0	0	0	0	0	0	0	0	0
0.05	0.131	0.124	0.171	0.162	0.154	0.203	0.193	0.184
0.1	0.262	0.248	0.342	0.324	0.308	0.406	0.386	0.368
0.15	0.394	0.373	0.515	0.487	0.463	0.61	0.58	0.553
0.2	0.528	0.5	0.688	0.652	0.62	0.815	0.775	0.74
0.25	0.663	0.629	0.861	0.817	0.778	1.019	0.971	0.926
0.3	0.8	0.76	1.035	0.983	0.937	1.222	1.166	1.113
0.35	0.937	0.891	1.207	1.149	1.096	1.423	1.359	1.299
0.4	1.073	1.023	1.376	1.312	1.253	1.619	1.548	1.481
0.45	1.207	1.152	1.54	1.47	1.406	1.807	1.729	1.657
0.5	1.335	1.277	1.695	1.62	1.552	1.983	1.9	1.822
0.55	1.455	1.395	1.836	1.758	1.686	2.141	2.053	1.971
0.6	1.562	1.5	1.957	1.878	1.804	2.274	2.184	2.099
0.65	1.651	1.588	2.052	1.972	1.898	2.373	2.283	2.196
0.7	1.714	1.653	2.111	2.033	1.959	2.429	2.339	2.254
0.75	1.743	1.685	2.124	2.049	1.979	2.428	2.342	2.26
0.8	1.728	1.675	2.078	2.01	1.945	2.355	2.277	2.201
0.85	1.66	1.614	1.96	1.901	1.845	2.197	2.129	2.063
0.9	1.529	1.493	1.757	1.712	1.669	1.937	1.884	1.833
0.95	1.323	1.302	1.453	1.427	1.402	1.554	1.524	1.494
1	1	1	1	1	1	1	1	1

The changes in heat transfer rate (Nusselt Number, Nu) are shown in Table 7. As seen in Table 7, heat transfer is greatest at the highest selected vales for the volume fraction of both nanoparticles. This means that the heat-carrying capacity of the hybrid nanofluid has risen. Table 7 shows that heat transfer increases with time.

**Table 7:** The variation in Nusselt number for various values of volume fractions of hybrid nanoparticles.

$\tau$	Nu $$\begin{gathered} \phi_{s1} = 0.00 \hfill \\ \phi_{s2} = 0.02 \hfill \\ \end{gathered}$$	Nu $$\begin{gathered} \phi_{s1} = 0.00 \hfill \\ \phi_{s2} = 0.04 \hfill \\ \end{gathered}$$	Nu $$\begin{gathered} \phi_{s1} = 0.02 \hfill \\ \phi_{s2} = 0.00 \hfill \\ \end{gathered}$$	Nu $$\begin{gathered} \phi_{s1} = 0.02 \hfill \\ \phi_{s2} = 0.02 \hfill \\ \end{gathered}$$	Nu $$\begin{gathered} \phi_{s1} = 0.02 \hfill \\ \phi_{s2} = 0.04 \hfill \\ \end{gathered}$$	Nu $$\begin{gathered} \phi_{s1} = 0.04 \hfill \\ \phi_{s2} = 0.00 \hfill \\ \end{gathered}$$	Nu $$\begin{gathered} \phi_{s1} = 0.04 \hfill \\ \phi_{s2} = 0.02 \hfill \\ \end{gathered}$$	Nu $$\begin{gathered} \phi_{s1} = 0.04 \hfill \\ \phi_{s2} = 0.04 \hfill \\ \end{gathered}$$
0.5	0.063	0.073	0.063	0.037	0.084	0.073	0.084	0.096
1.5	0.188	0.207	0.188	0.208	0.227	0.207	0.227	0.247
2	0.234	0.254	0.233	0.255	0.276	0.254	0.276	0.297

## Conclusion

The flow model Brinkman-type fluid with heat transfer and hybrid nanoparticles is the main topic of this work. Using the modified Fourier's law and the fractional derivatives operator, more specifically the Caputo fractional operator, the flow model after non-dimensionalization is generalised. The integral transformations are then used to solve the extended model precisely. The following are the study's main findings: The fractional model is more useful for physical processes because it permits curves to be fitted to exact solutions without changing physical parameters. The velocity increases as the Grashof number increases and decreases as the Hartman number increases, implying that the acquired results are genuine and accurate. Heat transfer is aided by the volume fractions of nanoparticles, and the temperature is higher for hybrid nanoparticles with the highest volume fraction.

## Data Availability

The database used and analysed during the current study are available from the corresponding author on reasonable request.

## List of symbols

$$\mu$$: Dynamic viscosity
$u$: The fluid velocity in the *x*-direction
$$\Theta$$: The temperature
$\beta_{C}$: The coefficient of concentration
$c_{p}$: The specific heat capacity of fluids
$D$: The mass diffusivity
$\rho$: The fluid density
$\beta_{r}$: The material parameter of Casson fluid
$\beta_{\Theta }$: The thermal expansion coefficient
$g$: The acceleration due to gravity
$k$: The thermal conductivity
