# Author Correction: Enhancement in heat transfer due to hybrid nanoparticles in MHD flow of Brinkman-type fluids using Caputo fractional derivatives

**DOI:** 10.1038/s41598-022-26239-2

**Published:** 2022-12-16

**Authors:** Nadeem Ahmad Sheikh, Dennis Ling Chuan Ching, Ilyas Khan, Hamzah bin Sakidin

**Affiliations:** 1 Fundamental and Applied Sciences Department, Universiti Teknologi PETRONAS, 32610 Seri Iskandar, Perak, Malaysia; 2 Department of Mathematics, College of Science Al-Zulf, Majmaah University, Al-Majmaah, 11952 Saudi Arabia

Correction to: *Scientific Reports* 10.1038/s41598-022-18110-1, published online 18 August 2022

The Funding section in the original version of this Article was incomplete.

“Yayasan Universiti Teknologi PETRONAS (YUTP), Cost Center 015LC0-278 received by D. L. C. C.”

now reads:

“Yayasan Universiti Teknologi PETRONAS (YUTP), Cost Center 015LC0-278 and NCRF 015MCO-030 received by D. L. C. C.”

The original Article has been corrected.

